# Molecularly Imprinted Polymer-Coated Inorganic Nanoparticles: Fabrication and Biomedical Applications

**DOI:** 10.3390/mi13091464

**Published:** 2022-09-03

**Authors:** Sinem Orbay, Ozgur Kocaturk, Rana Sanyal, Amitav Sanyal

**Affiliations:** 1 Institute of Biomedical Engineering, Bogazici University, Istanbul 34684, Turkey; 2 Department of Chemistry, Center for Life Sciences and Technologies, Bogazici University, Istanbul 34342, Turkey

**Keywords:** molecularly imprinted polymers, inorganic nanoparticles, biomedical applications

## Abstract

Molecularly imprinted polymers (MIPs) continue to gain increasing attention as functional materials due to their unique characteristics such as higher stability, simple preparation, robustness, better binding capacity, and low cost. In particular, MIP-coated inorganic nanoparticles have emerged as a promising platform for various biomedical applications ranging from drug delivery to bioimaging. The integration of MIPs with inorganic nanomaterials such as silica (SiO_2_), iron oxide (Fe_3_O_4_), gold (Au), silver (Ag), and quantum dots (QDs) combines several attributes from both components to yield highly multifunctional materials. These materials with a multicomponent hierarchical structure composed of an inorganic core and an imprinted polymer shell exhibit enhanced properties and new functionalities. This review aims to provide a general overview of key recent advances in the fabrication of MIPs-coated inorganic nanoparticles and highlight their biomedical applications, including drug delivery, biosensor, bioimaging, and bioseparation.

## 1. Introduction

**Figure 1:**
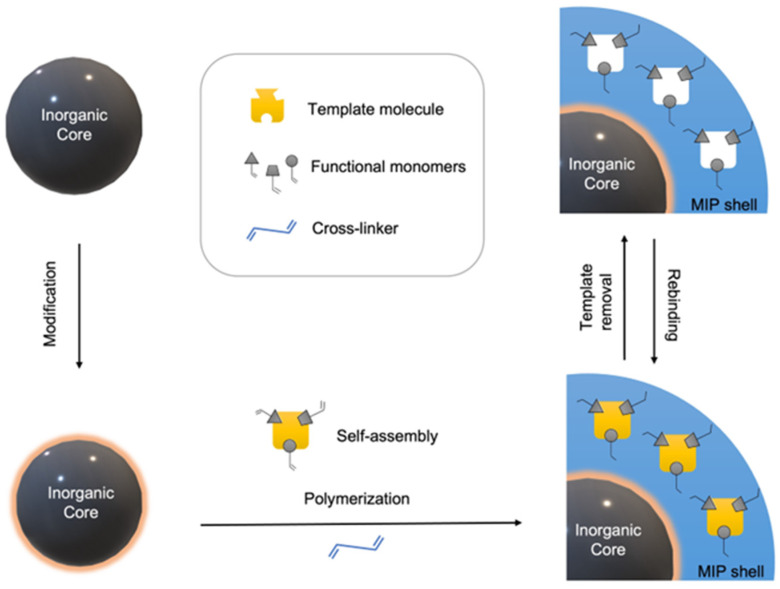
Illustration of molecularly imprinted polymer shell coating onto an inorganic core.

Many diagnostic and therapeutic platforms require selective recognition of a target molecule. Nature employs biological receptors such as enzymes [1], antibodies [2], or histones [3] as tools to recognize the respective antigens. However, using these biological components in clinical applications remains limited due to their physical and chemical instability and high cost. New synthetic materials have recently been proposed to provide more stable, durable, and cheaper alternatives [4]. Molecular imprinting is an approach that entails creating artificial recognition sites by polymerizing functional and cross-linking monomers in the presence of a target molecule (Figure 1) [5,6]. The preparation of MIPs first requires the formation of a pre-assembly between the template molecule and functional monomers via either covalent or non-covalent interactions. Then, cross-linkers arrest the temporary structure into a permanent one during the polymerization process to yield a three-dimensional polymeric network. After the polymerization, the template molecule is removed from the matrix to obtain the complimentary cavities. Herewith, the polymer carries the memory that can selectively recognize the targeted molecule.

Molecular imprinting has emerged as an attractive technology due to its remarkable features. These materials are highly stable, straightforward to synthesize, and possess superior mechanical properties. Furthermore, the method can be applied to a diverse range of target molecules, including amino acids [7], pharmaceuticals [8], pesticides [9], nucleotides [10], viruses [11], and cells [12]. Importantly, even when a biological receptor is unknown, imprinting technology can allow the generation of a tailor-made receptor-like material. These attractive features have expanded the application areas of MIPs from chemical separation [13,14], selective extraction [15], and catalysis [16,17] to molecular sensing [6,18,19,20,21,22], bioimaging [4], and drug delivery [23,24,25]. Given the ever-increasing importance of point-of-care testing devices [26,27], MIPs with microfluidic systems creates new devices and enables their utilization in point-of-care applications [28].

**Figure 2:**
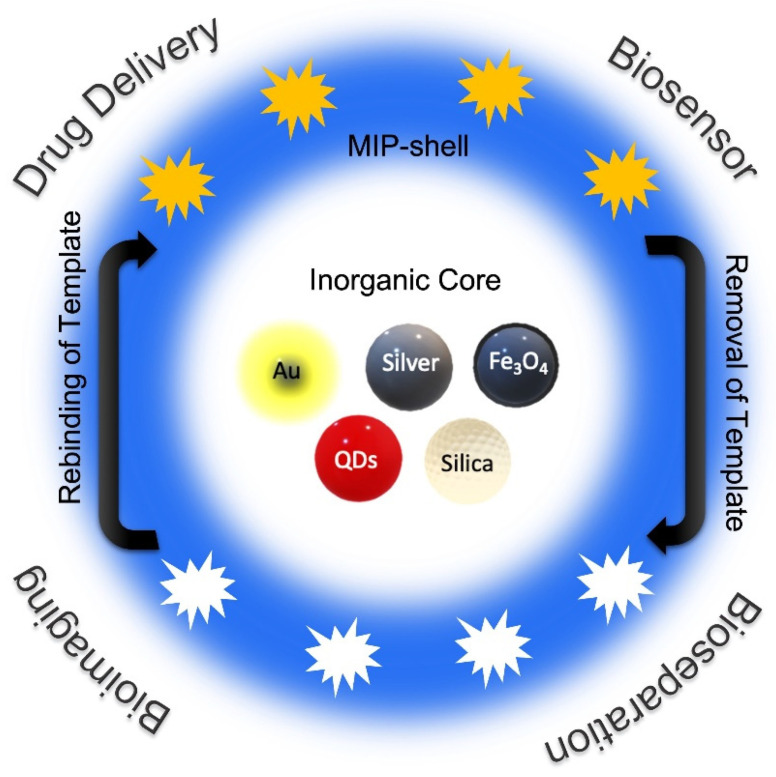
Illustration of various types of inorganic nanoparticles combined with MIP-shell and their possible biomedical applications.

Decades of research have demonstrated the superior performance of micro- and nanosized MIPs compared to their bulk macroscopic counterparts [29,30,31]. MIPs can be fabricated in various formats such as films [32], membranes [33], hydrogels [34], microparticles [35], multi-walled carbon nanotubes (MWCNTs) [36], or nanoparticles [37]. The overall performance of large and macroscopic bulk materials-based MIPs is compromised due to the heterogeneity of their analyte binding site and the poor accessibility of interior binding sites. Furthermore, removal of the template to obtain effective MIPs is also difficult. The large monolithic structures may also be difficult to integrate with a device or a signal transducer in a sensor. These problems are generally addressed by converting the bulk monolithic materials to microparticles by mechanical methods such as grinding. Compared to the bulk and microscale MIPs, nano-MIPs have several advantages, including a higher surface-to-volume ratio, accessibility of imprinted cavities due to easier template removal, improved recognition capabilities, and lower mass-transfer resistance [38]. Nano-MIPs can be produced by combining imprinted polymeric interface with inorganic nanoparticles or directly synthesizing MIP nanoparticles. Integration of MIPs with inorganic nanoparticles yields materials with predetermined particle sizes and distributions and provides flexibility in functionalization by choosing the appropriate nanomaterials. Furthermore, it was demonstrated that the sensitivity and selectivity obtained with MIP-coated inorganic nanoparticles are enhanced compared to the average response of its constituents. [39,40]. Gold (Au), silver (Ag), silica (SiO_2_), QDs, and iron oxide (Fe_3_O_4_) nanoparticles have been extensively used as core materials to support the surface imprinting of the template molecule on their polymeric shell [41]. Such a combination harnesses the intrinsic properties of the inorganic core and the tailored characteristics of the polymeric shell to obtain materials with unique functional characteristics suited for various biomedical applications (Figure 2).

This review aims to overview the leading fabrication methods for MIP-coated inorganic nanoparticles and highlight their biomedical applications. Recent examples in this field are presented by focusing on preparing MIP shell-coated inorganic core systems and their essential biomedical applications. First, a brief introduction of the synthetic approaches for the fabrication of MIPs is provided. This section is followed by a survey of the specific methodologies employed for obtaining MIPs using various inorganic nanoparticle cores. The methodology to obtain the MIP-based polymeric shell depends on the chemical composition of the inorganic core since the surface chemistry determines approaches for robust attachment of the polymeric shell. Finally, various examples of MIP-coated inorganic nanoparticles are discussed to highlight their biomedical applications, such as biosensing, drug delivery, imaging, and bioseparations. The overall aim of the review is to highlight the potential of MIP-coated nanoparticles and thus attract readers to further the design and implementation of such materials in addressing challenges in various areas of biomedical sciences.

## 2. MIP Synthesis

In the 1970s, Wulff and coworkers synthesized an organic polymeric material that was able to separate racemates. It was the primary demonstration of copolymerization of derivatives of a template molecule and a cross-linking monomer [42]. The interaction between the template molecule and the functional monomers classifies the imprinting approaches into two main classes: covalent and non-covalent. Covalent bonds between the template molecule and the suitable monomers require chemical cleavage that can reform during the rebinding process [43]. This method, however, remains limited due to the inability to perform reversible bonding and relatively harsh conditions for template removal. Mosbach and coworkers widely explored the non-covalent approach and successfully attempted to form a pre-polymerization complex between the template and functional monomers through non-covalent interactions [44,45]. In this bio-inspired approach, non-covalent interactions drive the self-assembly of monomers around the template to create the specific recognition site, as witnessed in various biomolecular recognition events in nature. While the method offers operational simplicity, this method tends to provide less homogenous rebinding sites than covalent ones.

Traditionally, MIPs were synthesized in bulk forms that require mechanical grinding to obtain polymeric particles with a larger surface area. The time-consuming procedure, irregular sizes within the range of 5–100 μm, and heterogeneous binding sites make this method undesirable. These shortcomings have directed the researchers to create monodisperse polymeric nanoparticles [46]. Due to their uniform geometric features, nanoparticles offer improved binding kinetics and easier template removal with reduced template leaching. In particular, MIPs with controlled size and physical forms are preferred for analytical applications. For example, regular shapes favor chromatographic and separation applications and homogenous binding sites are more suitable for binding assays.

Direct access to homogenous polymeric nanoparticles can be obtained using precipitation polymerization. Mosbach and coworkers cleverly employed this technique to synthesize MIP nanoparticles for the targets of theophylline and 17β-estradiol [47]. Later, many successful attempts were shown for various biomedical applications. For instance, Ruela et al. [48] demonstrated in vitro nicotine release and its permeability through the skin by using MIPs synthesized by precipitation polymerization, and Pardeshi et al. [49] explained how advantageous nano-MIPs prepared by the precipitation polymerization method is in chromatographic applications. While the method offers potential advantages, it requires the utilization of excess solvent. Due to the dilution factor during fabrication, MIPs with decreased selectivity is obtained. Emulsion polymerization offers another approach to synthesizing imprinted nanoparticles, usually in an oil-in-water system. This method provides better control over particle size distribution than precipitation polymerization. Tovar and coworkers [50] presented the direct non-covalent imprinting of L- or D-Boc-phenylalanine anilid using emulsion polymerization. They obtained nanoparticles with an approximate size of 200 nm in high yield. Nevertheless, surfactants or cosurfactants must be used for this imprinting method to stabilize the emulsion, which may result in interference with molecular recognition and contamination.

As an alternative approach, the MIP can be grown as a shell on a nanoparticle core. Such an approach yields MIP-coated core-shell nanoparticles, which will provide MIPs with uniform and predetermined particle sizes and create systems with increased multifunctionality like magnetic, optical, or semi-conductive, the later attributes arising from the inorganic core. Due to the aforementioned attractive characteristics, increasing interest in designing and fabricating MIPs based on inorganic nanomaterials has emerged. Combining traditional MIPs with nanostructures to create core-shell MIPs offers an opportunity to engineer materials with excellent properties by judicious choice of the nanoparticle core and the polymeric shell. Some of these advantages of core-shell MIPs over bulk MIPs are listed in Table 1. To date, silica (SiO_2_), QDs, gold (Au), and magnetic nanoparticles have been investigated as core materials. The imprinted polymeric shells have been prepared using precipitation, emulsion polymerization, or surface grafting methods.

## 3. Fabrication of Core-Shell Nanoparticles

Nanoparticles used as solid support needs surface anchoring of the polymeric materials to provide robust adherence to the MIP shell. Different approaches for ensuring such integration of the polymeric component are required depending on the chemical composition of the inorganic core materials. One such approach involves surface modification of the inorganic core with heterobifunctional molecules as an adlayer that chemically binds to the inorganic surface from one end and has the other end available for attachment with the organic polymeric component. Alternatively, the polymeric component may contain functional groups that chemically attach to the inorganic surface. The sections below provide illustrative examples of approaches employed for different inorganic cores.

It should be noted that thorough characterization of core-shell nanoparticles is an important requirement to ensure understanding and validation of the recognition process, as well as warrant reproducibility of the performance of obtained MIP-coated nanoparticles. Different techniques can characterize the physical properties of fabricated nanoparticles. While dynamic light scattering (DLS), electron microscopy, or X-ray diffraction methods provide information about the size of nanoparticles, scanning electron microscopy (SEM) gives information about the surface topography of the nanoparticles. Transmission electron microscopy (TEM) is perhaps the suitable tool to visualize the presence of the polymeric shell around the inorganic core. Additionally, the surface composition of the nanoparticles was characterized by ultraviolet-visible spectroscopy (UV-vis), X-ray photoelectron spectroscopy (XPS), or Fourier transform infrared spectroscopy (FTIR).

### 3.1. Silica (SiO_2_) NPs

**Figure 3:**
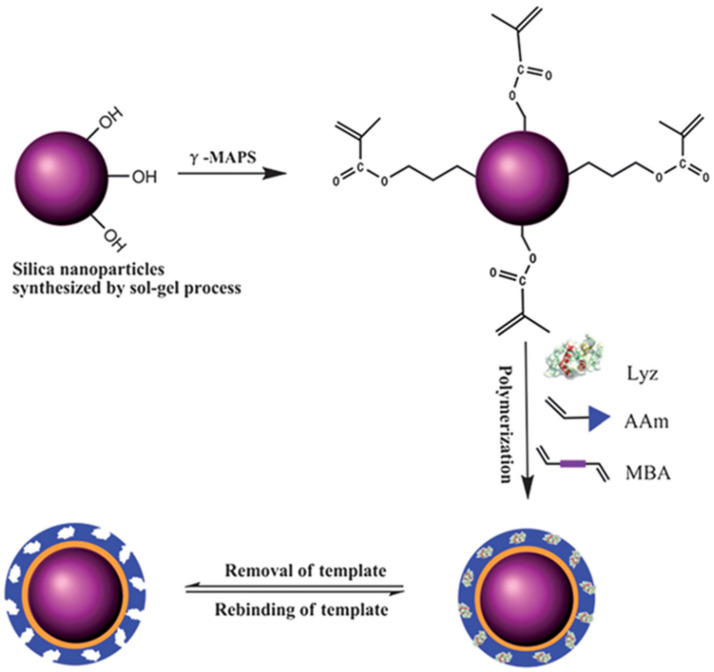
General scheme of surface Lyz imprinting over the surface of silica nanoparticles. Reproduced with permission from [52].

Silica nanoparticles are widely used core material due to their well-established synthesis process (mainly the Stöber method), flexible chemistry, stability under certain conditions, and apparent biocompatibility [51]. Silica nanoparticles possess hydroxyl groups on their surfaces, which react with alkoxysilane-containing molecules to provide grafting sites for the polymer shell. Using appropriately functionalized alkoxysilane derivatives, silica nanoparticles can be functionalized with vinylic, isocyanate, or amine groups. After that, the imprinting of the target analyte is undertaken using the polymerization of functional monomers and cross-linkers. For example, Chen and coworkers [52] used silica nanoparticles with a diameter of 400 nm as a core substrate for lysozyme (Lyz) recognition. Tetraethoxysilane (TEOS) was used for the synthesis of nanoparticles, followed by their modification with γ-methacryloxypropyltrimethoxysilane (γ-MAPS) to introduce the vinyl groups on the surface. A thin film MIP layer was fabricated on these vinyl-modified nanoparticles by the surface imprinting of lysozyme using acrylamide (AAm) and *N*,*N*’-methylenebisacrylamide (MBA) as monomer and cross-linker, respectively (Figure 3).

Zhang and coworkers presented another study on the extraction of sulfonamides [53]. Silica nanoparticles of diameter 80 nm were first functionalized with 3-aminopropyltriethoxysilane (APTES) to install amine groups on the surface. Subsequently, vinyl groups were introduced using acryloyl chloride (CH_2_CHCOCl)-based modification. The vinyl end groups were co-polymerized with the functional monomer (acrylamide) and the cross-linker (EGDMA) in the presence of the target molecule (sulfamethoxazole). The resulting nanoparticle with a MIP shell demonstrated highly accessible binding sites.

In 2010, Chang et al. reported the preparation of core-shell MIPs by combining the reversible addition-fragmentation chain transfer polymerization (RAFT) and click chemistry [54]. Azide-modified silica nanoparticles and alkyne terminated RAFT chain transfer agent (CTA) were synthesized separately following click reaction between silica and RAFT agent. A thin MIP film with a thickness of 2.27 nm was grafted onto the silica surface to create recognition sites for 2,4-dichlorophenol. This hybrid design showed high affinity, better rebinding kinetics, and selectivity toward target molecule.

**Figure 4:**
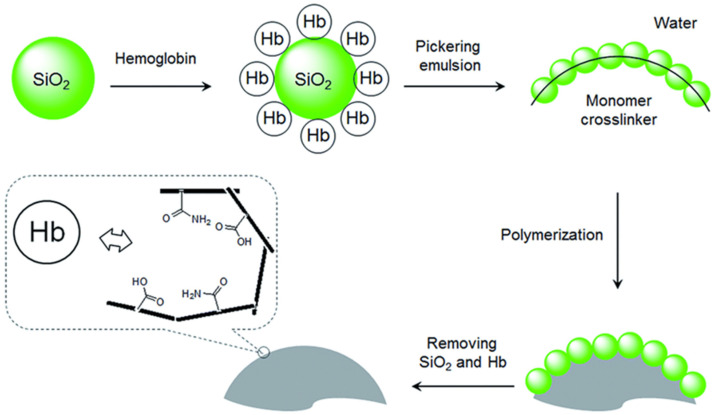
Demonstration of Pickering emulsion procedure for protein imprinting. Reproduced with permission from [55].

As a different approach, Pickering emulsion polymerization using protein-modified silica beads was reported by Ye and coworkers [55]. Silica particles were coated with the target protein hemoglobin (Hb) and utilized to stabilize a Pickering emulsion. The oil phase comprised the cross-linker and monomers, which were polymerized through free radical polymerization. To obtain template imprinted recognition sites into the polymeric matrix, Hb-coated silica nanoparticles were removed. This approach for the MIP particles creates a well-controlled structure with larger pore size. High selectivity with faster recognition time was obtained since the recognition sites are accessible on the surface, as illustrated in Figure 4.

### 3.2. Magnetic NPs

Magnetic fields can manipulate magnetic nanoparticles composed of iron, cobalt, nickel, or alloy. Iron oxide (Fe_3_O_4_) nanoparticles are favorably used in many biological applications since they have low toxicity and a low-cost profile [56]. Most of the studies in the field of MIP-coated magnetic nanoparticles utilize Fe_3_O_4_ nanoparticles. The external magnetic field easily separates the MIP-coated Fe_3_O_4_ from the solution, thus eliminating centrifugation and filtration procedures in recognition, extraction, or separation applications.

A primary approach for obtaining a MIP-coated Fe_3_O_4_ nanoparticle utilizes the “grafting-from” method, where imprinted polymers are grown from the nanoparticle’s surface using contemporary polymerization methods such as atom transfer radical polymerization (ATRP) and reversible addition-fragmentation chain transfer polymerization (RAFT). Apart from being tolerant to a wide range of functional groups, these polymerization techniques are well-controlled, thus allowing homogenous chain growth to yield functional coatings with uniform thickness. Thus, the grafting-from technique offers a powerful method for obtaining polymer-coated nanoparticles [57,58], and its utilization in obtaining MIP-coated nanomaterials continues to grow.

**Figure 5:**
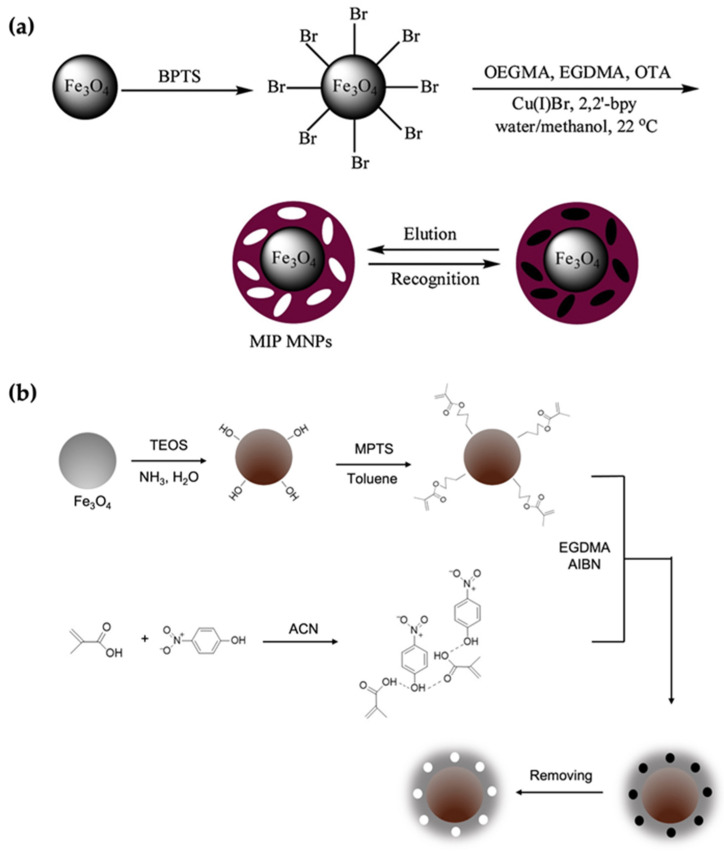
(**a**,**b**) The formation mechanisms of the surface imprinting process use the “grafting-from” technique. Reproduced with permission from [59,60], respectively.

In a recent example, Sahin and coworkers used the “grafting-from” approach to extract the carcinogenic mycotoxin ochratoxin-A [59] selectively. First, Fe_3_O_4_ nanoparticles were functionalized with (3-bromopropyl) trimethoxy silane (BPTS) to install -Br groups as radical initiators on the surface (Figure 5a). The MIP synthesis was performed in the presence of oligo (ethylene glycol) monomethyl ether methacrylate as a monomer, ethylene glycol dimetachrylate as a cross-linker, and the template molecule via surface-initiated ATRP. A polymerized MIP shell with an average thickness of 18 nm was obtained. According to their experimental results, the MIP-coated magnetic nanoparticles indicated fast adsorption, perfect selectivity, and high affinity toward the target molecule.

Generally, the inorganic nanoparticles are modified with a silica layer to provide facile and robust surface modifications, increase biocompatibility, avoid the leakage of metal ions, and prevent their surface oxidation. This approach has been extensively used for modifying magnetic nanoparticles before their utilization as a core. It should be noted that the additional silica layer leads to a decreased magnetization of Fe_3_O_4,_ but it is still sufficient for applications [61]. For example, Liu et al. proposed a novel Fe_3_O_4_@SiO_2_ @NH_2_ nanoparticle with a pefloxacin mesylate (PEF-M) imprinted shell [62]. The fabrication of this novel nanoparticle started with TEOS functionalization in the presence of ammonium hydroxide to obtain a silica layer onto the Fe_3_O_4_ nanoparticles. Later, the amino groups were introduced onto the Fe_3_O_4_@SiO_2_ by treatment with ammonium hydroxide and APTES. This step was followed by the preparation of imprinted polymer shells by ATRP. Another example was reported by Mehdinia et al. [60], where authors utilized the grafting method for the selective recognition of 4-nitrophenol (4-NP). They first modified the magnetic nanoparticles with tetraethyl orthosilicate (TEOS) and 3-methacryloxypropyl trimethoxy silane (MPTS). Then the surface was used for grafting a polymeric layer composed of the functional monomer methacrylic acid (MAA) and cross-linker ethylene glycol dimethacrylate (EGDMA). They investigated the nanoparticles’ adsorption capacity and selectivity performance by comparison with non-imprinted polymers (Figure 5b).

### 3.3. Quantum Dots (QDs)

Quantum dots (QDs) are fluorescent semiconductor nanomaterials that possess unique optoelectronic characteristics [63]. QDs have been widely used in bioimaging applications for disease diagnosis due to their distinctive optical features. Additionally, they have been utilized as luminescent probes in bioassays or as bio/chemosensors [64]. Hence, incorporating a MIP shell on QDs has grabbed the attention of researchers to explore new sensing strategies. The fabrication methods for preparing MIP@QDs can be divided into three categories [65].

**Figure 6:**
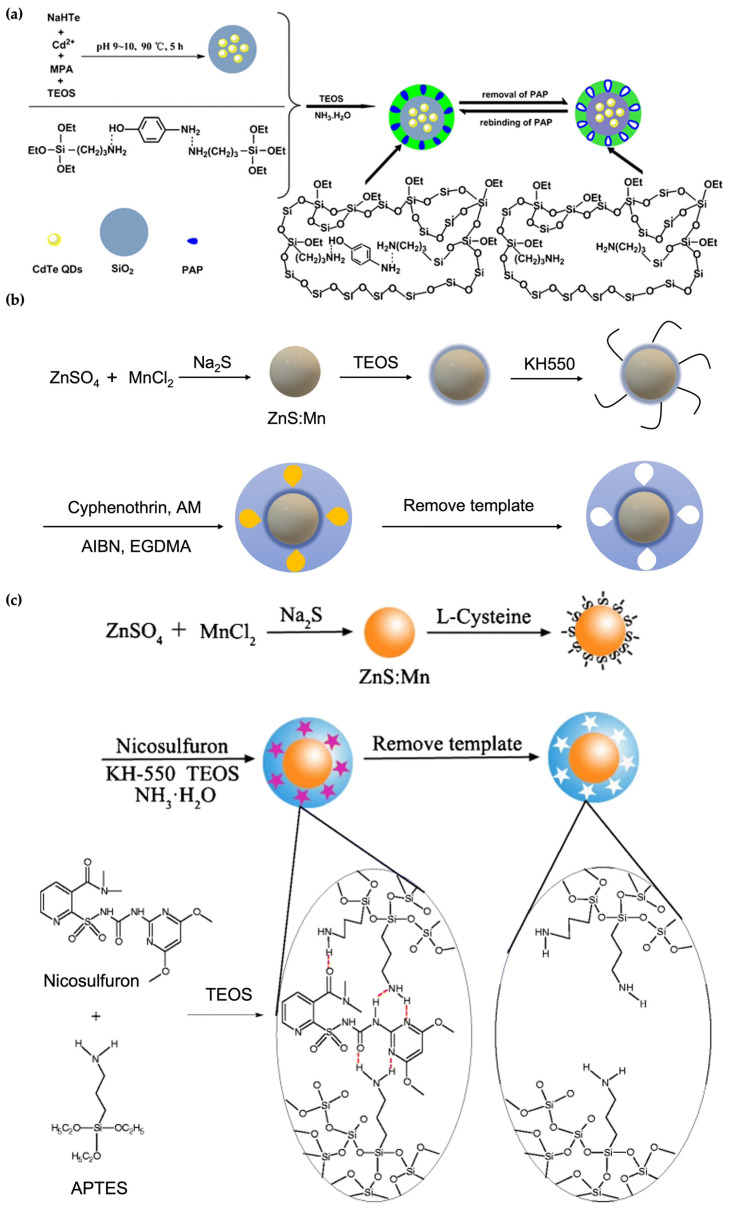
Demonstration of different strategies for preparing MIP@QDs. (**a**) Silica coating onto QDs, (**b**) inorganic–organic MIP@QDs, and (**c**) organic MIP@QDs. Reproduced with permission from [66,68,69] respectively.

In the first approach, a silica coating is introduced onto the QDs. This method takes advantage of silica’s stability, nontoxicity, and biocompatibility. Using such a modification, Lu et al. designed a sensor system for recognizing p-aminophenol (PAP) by grafting a MIP layer onto silica-coated CdTe QDs [66]. They used a one-pot approach [67] for the synthesis of CdTe@SiO_2_, which allows first the CdTe QDs formation, followed by the silica adhering in the presence of TEOS in the same reactor (Figure 6a). The introduction of the silica shell creates hydroxyl groups onto the nanoparticle surface, which are subsequently utilized in the imprinting step. APTES is used as a functional monomer, and a silanization reaction was employed for the production of the imprinted polysiloxane shell.

The second approach addresses overcoming the drawback of silica’s limited modifiable chemical functionalities. Since organic polymers hold a more extensive selection of functional groups, combining inorganic and organic compounds for the MIP@QDs is desirable. In these hybrid systems, QDs can act as either a sensing material or a supporting material in the core of the final core-shell nanoparticles. For example, a cyphenothrin imprinted polymer was synthesized onto a QD which acts as a fluorescence probe for pesticide detection [68]. As illustrated in Figure 6b, the first step involved the synthesis of Mn-doped ZnS QDs, with subsequent stabilization with a silica layer by hydrolysis of TEOS. Amine groups were introduced onto the surface by silanization with APTES. Surface imprinting on the amino-modified QDs was done in the presence of acrylamide (AM) as the functional monomer, ethylene glycol dimetachrylate (EGDMA) as the cross-linker, and the target molecule (cyphenothrin). Quenching of the fluorescence of the MIP-coated QDs upon interaction with cyphenothrin enables selective sensing of this pesticide.

The third strategy generally employed for the fabrication of MIP@QDs involves directly coating the polymeric layer on the inorganic core. Even though silica is mainly used in this field and the QDs@organicMIPs are reported less, the approach offers certain advantages. For example, Chen and coworkers compared QD-based MIPs consisting of imprinted silica versus an imprinted organic polymer-based shell. Experimental results showed that the acrylate-based MIP@QDs exhibited better recognition than the silica-based nanomaterial [70]. In addition, the direct incorporation of polymeric shell decreases the number of preparation steps and, as a result, shortens the fabrication time. Later on, another organic MIP@QDs example was demonstrated by Ren et al. [69]. They presented the combination of inorganic (Mn-doped ZnS QDs)-organic MIPs to determine nicosulfuron in water samples. The synthesis process is comprised of first, the fabrication of Mn-doped ZnS QDs, and second, the direct surface imprinting of the polymers onto the nanoparticle Figure 6c. The resulting QDs nanoparticles had a high selectivity toward nicosulfuron and a substantial fluorescence property.

### 3.4. Gold (Au) and Silver (Ag) NPs

Gold (Au) nanoparticles are among the most widely used core materials for the fabrication of inorganic nanoparticle-based MIPs since they possess a larger surface area, conductivity, high surface energy, and a characteristic surface plasmon resonance [71]. These features make Au nanoparticles desirable in the applications of biosensing and diagnostics. Hence, various sensor designs have been investigated by combining Au nanoparticles with MIPs to obtain analytical platforms.

**Figure 7:**
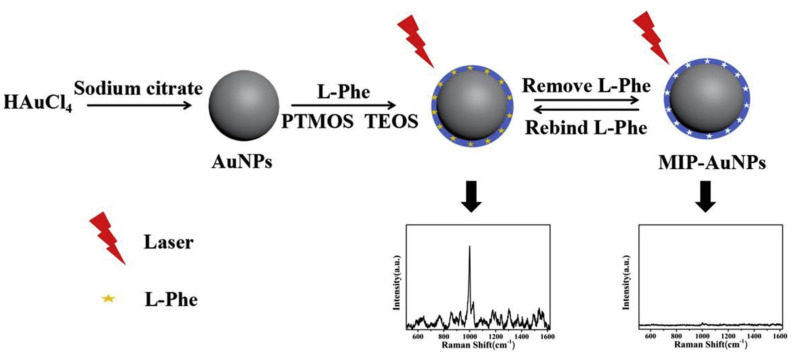
General scheme for the synthesis of the MIP@AuNPs, and SERS signal of MIP@AuNPs before and after the elution of the target molecule. Reproduced with permission from [73].

For example, in 2015, Yang et al. designed a MIP-coated Au nanoparticle system that was covered onto an electrode to build an electrochemical sensor [72]. First, the Au nanoparticles were synthesized by reducing HAuCl_4_. Then, an ionic liquid (IL) was self-assembled onto the nanoparticle surface to be further used as a monomer to synthesize MIPs in the presence of dimetridazole as the template, EGDMA as the cross-linker, and AIBN as the initiator. The resultant core-shell nanoparticle system was coated onto the carbon and graphene-modified glassy carbon electrode surface to build a sensor. Recently, another example of the MIP-coated Au nanoparticles for the selective recognition and quantification of L-phenylalanine (L-Phe) was demonstrated by Zhou et al. [73]. MIP@Au nanoparticles were utilized as a surface-enhanced Raman scattering (SERS) interface. After preparing the Au nanoparticles, the authors constructed the imprinted polymer layer onto the nanoparticles by sol-gel and surface imprinting techniques (Figure 7). The polymerization was performed with tetraethyl orthosilicate as the cross-linker and phenyltrimethoxysilane as the functional monomer. When removal of the template molecule from the MIP shell was completed, rebinding was observed according to the obtained SERS signals.

**Figure 8:**
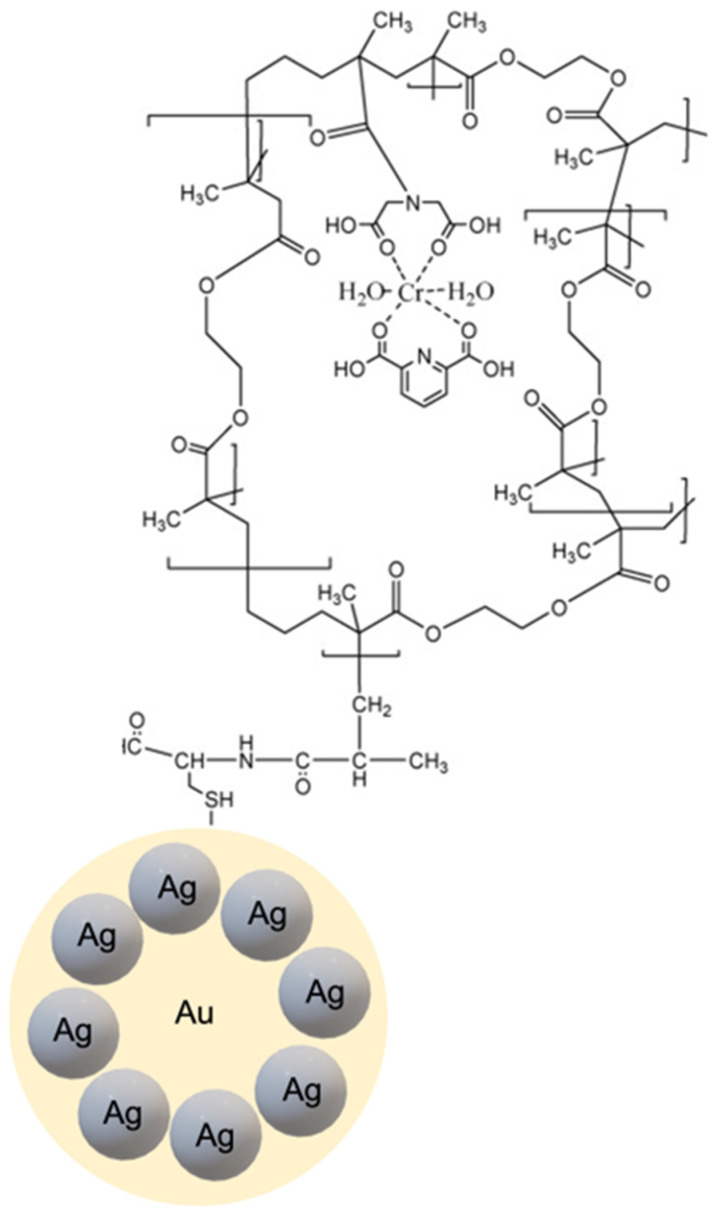
Representation of Au-Ag nanoclusters with shell based on DPA template imprinting. Reproduced with permission from [74].

In a different approach, Gultekin et al. [74] employed a nanocluster system composed of Au and silver (Ag) nanoparticles for the detection of Bacillus cereus spores through recognition of dipicolinic acid (DPA), a characteristic compound present in the Bacillus spores. A thiol-exchange capping method was utilized to synthesize gold-silver nanocomposite-based core material. Polymerizable methacryloylamidocysteine (MAC) was attached to the gold nanoparticle surface, followed by the addition of silver salt (AgNO_3_) to obtain the Au-Ag nanocluster. To construct the MIP shell, methacryloyl-activated nanoclusters were subjected to photopolymerization after adding the metal-chelate (MAIDA-Cr(III)/DPA) monomer, EDMA cross-linking, and initiator. The Au-Ag nanocluster-based MIPs were obtained after removing the template molecules using acidic conditions (Figure 8). Fluorescence quenching of the Au-Ag nanocluster MIP was used as a modality for detecting DPA.

**Figure 9:**
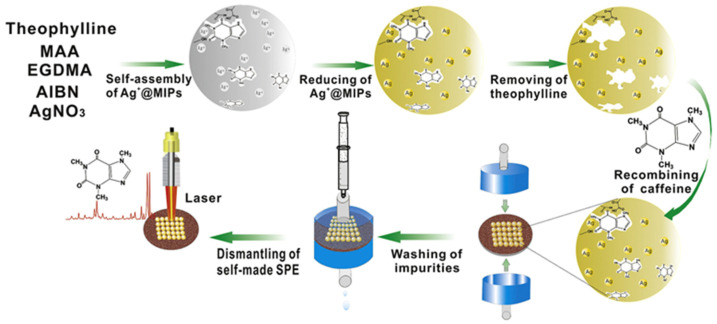
Synthesis of theophylline imprinted AgNPs, and subsequent SPE of caffeine before SERS detection. Reproduced with permission from [75].

The utilization of Ag nanoparticles plays a vital role in biomedical applications among metallic nanoparticles. Using them as a SERS substrate is widely used and well-established in the literature [30]. For the fabrication of a MIP-based SERS sensor, Hu et al. developed theophylline-imprinted polymer-loaded Ag nanoparticles [75]. They used precipitation polymerization to form the Ag@MIP system to be further used as a solid phase extraction (SPE) agent and SERS substrate for the single-step extraction and the quantification of caffeine. Theophylline was chosen as the dummy template. The polymeric layer was formed in excess acetonitrile with MAA as the functional monomer and EGDMA as the cross-linker (Figure 9). The overall time for the detection and separation of caffeine was determined as 23 min with a detection limit of 100 ng/L.

## 4. Biomedical Applications of Core-Shell MIPs

### 4.1. Biosensors

Selective recognition of a target molecule is a requisite for a biosensing system. Recognition of a specific biomarker, protein, or antibody is required in diagnostic applications, and such recognition is accomplished using biomolecules such as proteins and antibodies. As discussed in the introduction, MIPs offer a synthetic alternative to these natural detection elements. A literature survey indicates that core-shell MIPs containing an inorganic nanoparticle core enable the fabrication of a wide range of analytical sensors. The output detection signal is sorted as an electrochemical, optical, or piezoelectric response depending on the core material.

Magnetic nanoparticles for electrochemical sensing have been widely employed for diagnostic applications. In the context of molecular imprinting-based sensing, Cao and coworkers proposed an approach for hemoglobin detection in clinical diagnostics [76]. Hemoglobin (Hb) is an important biomarker that gives information regarding many clinical diseases such as hypertension, pulmonary diseases, and leukemia. Thus, developing methods for rapid and accurate quantification is essential. Their sensor design was based on Fe_3_O_4_@SiO_2_ composite nanoparticles with a MIP layer. The Hb oxidation measurements at the glassy carbon electrode surface allowed the determination of Hb with a response time of 7 min. The authors also tested the sensor using whole blood samples with recoveries between 95.7% and 105%.

As another application, magnetic core-shell nanoparticles were prepared for the selective determination of microorganisms to address antimicrobial resistance by Mishra and coworkers [77]. They used Fe_3_O_4_ nanoparticles as the core material, which was surface modified with 3-methacryloxypropyltrimethoxysilane (MPS). The imprinted polymer was synthesized onto the core material using pyocyanin as the template molecule, which is directly related to *Pseudomonas* infections. Authors utilized the core-shell design to detect bacteria in complex biological samples. The binding capacity and the imprinting factor of pyocyanin to the MIP-coated nanoparticle was found to be 2.5 mg/g and 5, respectively.

Recently, another electrochemical sensor example for detecting tumor necrosis factor-alpha (TNF-α) was reported [78]. Neonatal sepsis is a bloodstream infection encountered in newborn infants and causes neonatal deaths [79]. The TNF-α level is used as an indication of this condition. Previously used methods for neonatal sepsis diagnosis have drawbacks such as higher cost, time-consuming features, or difficulty in operations [80,81]. The proposed platform used a Fe_3_O_4_@SiO_2_ -modified screen-printed electrode that requires less sample volume from 50 to 100 μL to analyze the sample. The instant detection of the biomarker was achieved with a low detection limit of 0.01 pM.

MIP@QDs nanomaterials offer unique optical superiority, which makes them attractive components in the fabrication of devices for sensing applications. Mehrzad-Samarin et al. designed MIP-based graphene QDs for fluorescence-based detection of metronidazole [82]. Metronidazole is an antibacterial drug that is generally used for the treatment of acne diseases. This sensor system was proposed to monitor drug resistance caused by long-term and uncontrolled usage. A proportional fluorescence quenching in the presence of the drug was observed.

**Figure 10:**
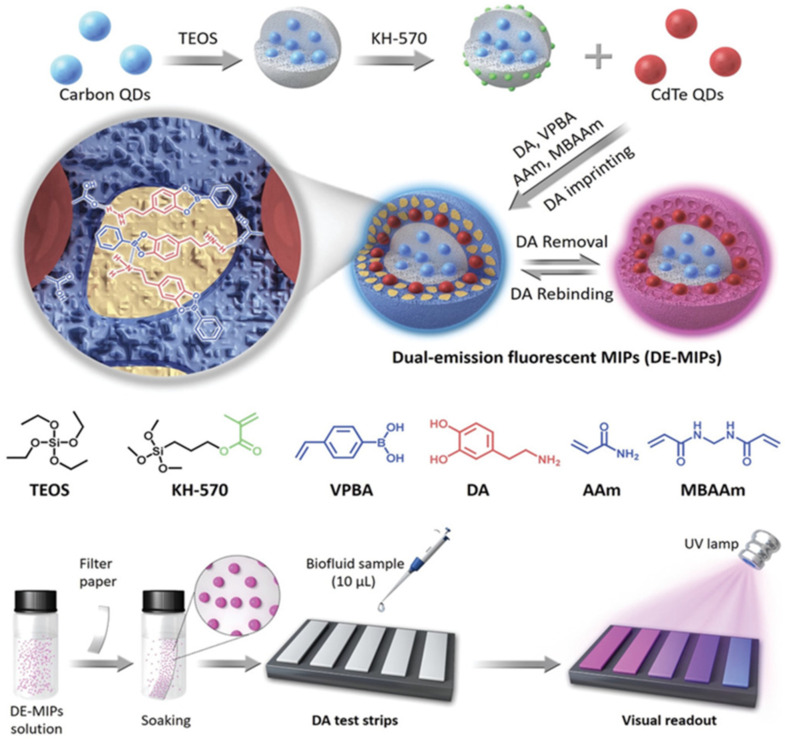
General scheme of generation and operation of the QDs@MIP. Reproduced with permission from [83].

The fluorescence sensor systems use an optical readout method. It is desirable to have a handheld readout device to give faster results and provide portability, as Wang et al. presented [83]. They introduced a MIP-based-QDs approach for dopamine detection in tiny amounts of fluids. Dopamine is a neurotransmitter correlated to several pathological disorders such as Parkinson’s, schizophrenia, and hyperactivity disorders [84]. Their system is a paper-based assay that enables dopamine detection in 10 μL of serum within the linear range of 0–1.2 × 10^−6^ M. MIP shell fabricated using a boronic acid unit which has a high affinity for dopamine was coated onto the dual emission QDs to achieve visual verification of the presence of dopamine using exposure to UV light, and the detection limit was calculated as (100–150) × 10^−9^ M (Figure 10).

Another dopamine detection system was introduced by Shi and coworkers [85]. A MIP-based voltammetric sensor was fabricated using Au nanoparticles covered with biocompatible porous SiO_2_. A MIP polymer layer was assembled onto the nanoparticle through the hydrogen bonding and π–π interactions between dopamine molecules and the silane-based monomers. Cyclic voltammetry measurements were carried out using the glassy carbon electrode for the detection of dopamine in the range from 4 × 10^−8^ to 5 × 10^−5^ M within the detection limit of 2 × 10^−8^ M. They tested the sensor design in human urine samples which performed as a versatile tool for DA determination.

Gold nanoparticles are among the most widely used substrates in SERS-based sensing. Using gold nanoparticle-based MIP, Li and coworkers designed an antibody-free immunoassay [86]. The sensor system was used to precisely determine carcinoembryonic antigen (CEA), a protein typically found in deficient levels in the blood, and increased level is associated with certain types of cancer. MIP preparation was done using 4-vinylbenzeneboronic acid (VPBA) as the functional monomer and ethylene glycol dimetachrylate (EGDMA) as the cross-linking agent. The resulting sensor, also called SERS-tag, was used to quantify CEA in spiked serum within the detection limit of 0.1 ng/mL. For a quick overview, the above examples are summarized in Table 2.

### 4.2. Drug Delivery

The imprinting technology is of interest in pharmaceuticals because the polymer-nanoparticle composites can act as versatile drug delivery vehicles. Polymer based nanocarriers have been extensively employed in delivery of imaging agents and therapeutically active molecules [87]. It has been demonstrated that the MIP-based drug delivery systems enhanced drug efficiency with desired controlled release and decreased side effects due to better drug encapsulation [23,88,89]. Surface properties of the inorganic material have an important role in determining the overall biocompatibility; therefore, surface modifications can deliver MIP-based core-shell systems suitable for in vivo studies.

**Figure 11:**
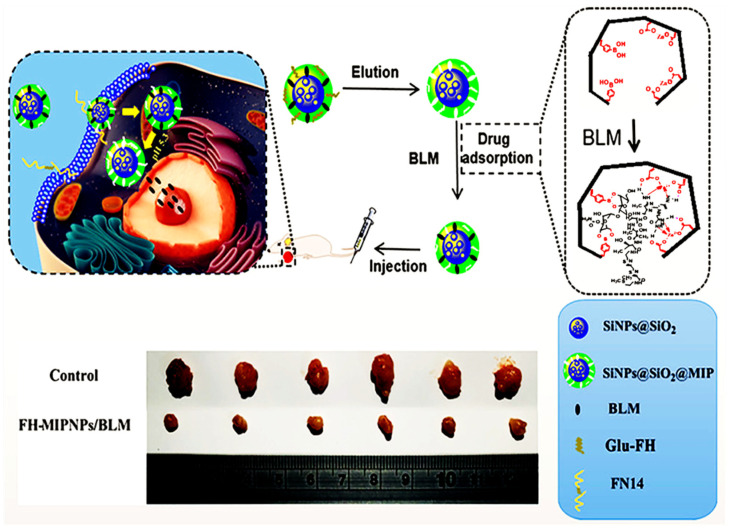
General illustration of preparation and application of silane-modified MIP@SiO_2_. Reproduced with permission from [90].

Jia et al. [90] presented a MIP@nanoparticle design for the dual templates; bleomycin (BLM) and 71–80 peptide of human fibroblast growth-factor-inducible 14 modified with glucose (Glu-FH). They first synthesized the SiO_2_ nanoparticles and modified them by silanization. Dual templates-imprinted polymers covered the surface of nanoparticles to be used for both targeting and the drug delivery for pancreatic cancer BxPC-3 cells. Targeting and the drug release performances of the design were first confirmed by in vitro experiments, followed by the in vivo tumor treatment. The results proved these MIP-coated nanoparticles’ low toxicity and tumor growth inhibition capacity (Figure 11).

Zavareh, Hashemi-Moghaddam, and coworkers investigated the usage of Fe_3_O_4_ nanoparticles in the drug delivery system in two parallel studies [91,92]. They worked with the breast tumor-induced mouse models and delivered the doxorubicin (DOX) and 5-fluorouracil (5-FU) drugs to the tumor sites. Both studies used dopamine, which can be self-polymerized, to adhere the polydopamine layer onto the Fe_3_O_4_ core. The mouse models used an external magnetic field to deliver the drugs. Recently, Nerantzaki et al. proposed the DOX-loaded molecularly imprinted polymer adhering with Fe_3_O_4_ nanoparticles [93]. They designed by using a biotinylated surface since biotin divides the cancer cells and thus, increases the drug uptake.

**Figure 12:**
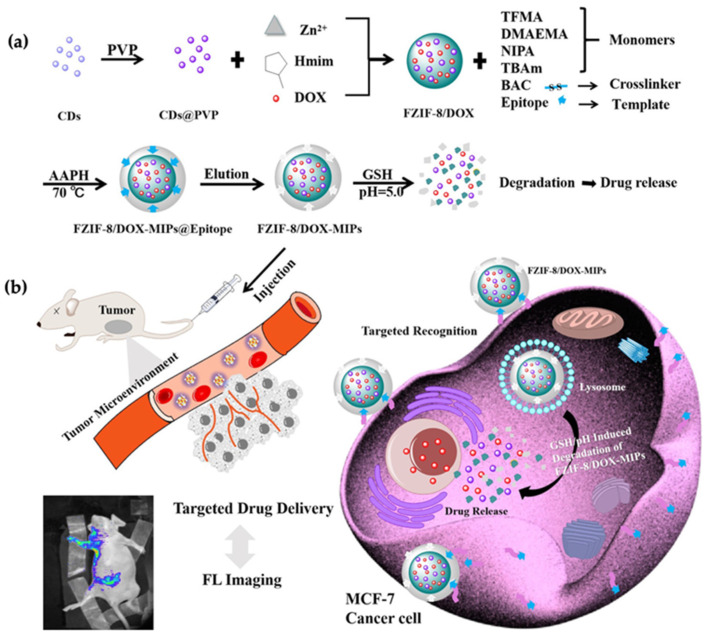
(**a**) Synthesis of tumor-sensitive degradable DOX-loaded MIP nanoparticles, (**b**) illustration of targeted imaging and drug delivery. Reproduced with permission from [94].

In another example, a dual stimulative drug delivery system was developed using carbon-based QDs (CQDs) [94]. Zhang and coworkers synthesized the tumor-sensitive biodegradable DOX-loaded MIP particles. The particles targeted the MCF-7 cancer cells, and the particle degradation was induced by low pH and high glutathione levels. The preparation of MIPs@nanoparticles was done with an epitope of the CD59 cell membrane glycoprotein and DOX as templates, and the high concentration of glutathione and weak acidic environment due to the cancer cells triggered DOX release (Figure 12a). They employed fluorescent zeolitic imidazolate framework-8 (FZIF-8) as the framework since its fully biodegradable nature in an acidic environment. The fluorescence intensity of MIP@nanoparticles was found ~4 times higher than the control group, which indicated enhanced adsorption and strong selectivity. Additionally, the viability of MCF-7 cells was dramatically lower after the treatment with the FZIF-8/DOX MIP nanoparticles, with 20% survival after 72 h compared to the control trials. In vivo experiments showed that tumor volume in mice was found to be smaller after being treated with FZIF-8/DOX MIP nanoparticles (Figure 12b). Most importantly, they did not observe significant apoptosis in normal cells, which showed successful selective targeting.

### 4.3. Bioimaging

Bioimaging plays a crucial role in life-science studies since it enables scientists to visualize the biological activities to analyze molecules, cells, or tissues. Bioimaging involves the determination of the targeted biological entities selectively but at the same time with the information related to their spatial localization. Integrating inorganic nanoparticles and MIPs elevate the bioimaging performances of functional nanomaterials. The approach is attractive since the preparation method is usually adaptable for any targeting, the biocompatibility of core-nanoparticles can be enhanced with surface modifications, and the desired core material can be employed.

**Figure 13:**
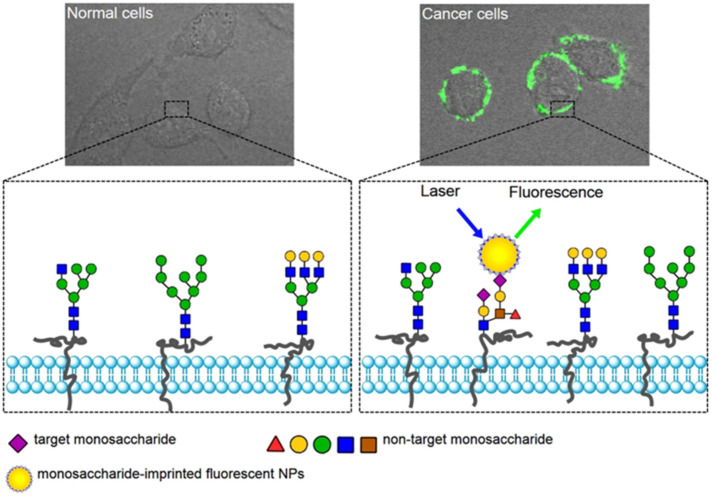
Illustration of the targeting and imaging of cancer cells with MIP@FTC@SiO_2_ nanoparticles. Reproduced with permission from [95].

When the bioimaging and drug delivery modalities are combined, the platform becomes much more desirable since additional information such as how long MIP-coated nanoparticles stay in the bloodstream and if they target the site of interest can be obtained. Such an approach was demonstrated by Liu and coworkers, where authors designed monosaccharide-imprinted fluorescence nanoparticles for both the targeting and imaging of human hepatoma carcinoma cells (HepG-2) and mammary cancer cells (MCF-7) (Figure 13) [95]. They used fluorescein isothiocyanate (FITC)-doped SiO_2_ nanoparticles with an imprinted shell. The SiO_2_ nanoparticles were functionalized with boronic acid to create boronate affinity. It was confirmed that boronate affinity-based approaches enable efficient and controllable preparation of antibody mimics for monosaccharides. The same boronate-affinity system was also used in another study of the same group [96]. This time, Ag@ SiO_2_ nanoparticles were functionalized with boronic acid and investigated in SERS imaging. Sialic acid imprinted polymers recognized the cancer cells and tissues where the sialic acid overexpressed.

**Figure 14:**
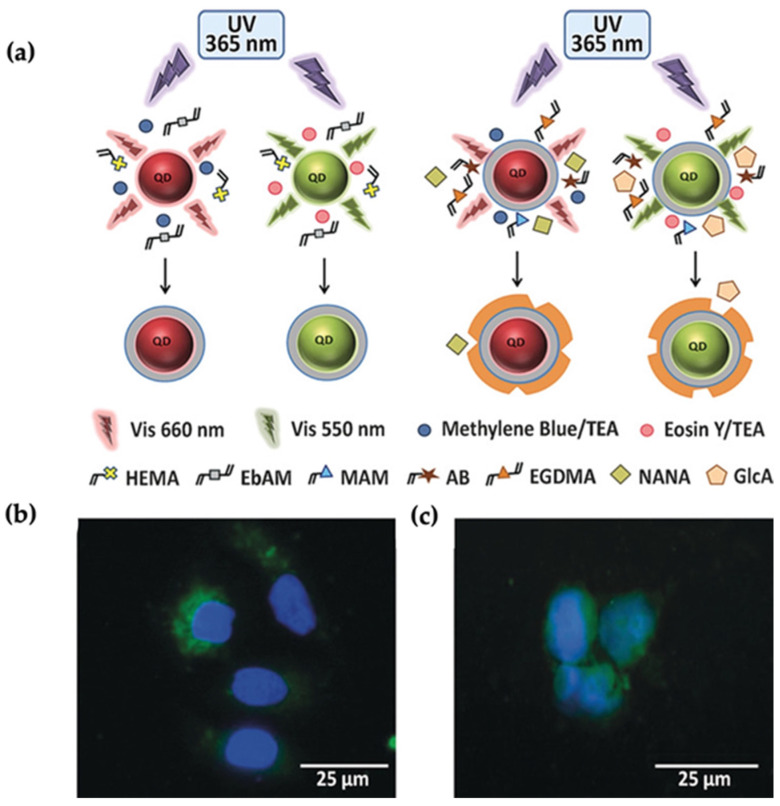
(**a**) Demonstration of red and green light emitting QDs excited by UV and MIP shell around the particles. (**b**,**c**) Staining of keratinocytes, (**b**) KU812 cells, (**c**) with MIP@QDs Reproduce with permission from [98].

Apart from the tumor glycan targeting, Haupt and coworkers designed fluorescent nanoMIPs to target and image the normal cell and tissues for the first time [97]. They demonstrated the fluorescents nanoMIPs prepared by precipitation polymerization in the presence of polymerizable rhodamine dye. Fluorescently labeled polymers imprinted with glucuronic acid (GlcA) to be used for the identification of hyaluronic acid of keratinocytes; therefore, the red-fluorescent nanoMIPs were localized on the cell surface and used as an imaging tool for human keratinocyte cells and adult skin tissue. Later the same group proposed a core-shell design (MIP-coated QDs) for the imaging of multiple targets; cell membrane glycans, glucuronic acid (GlcA), and N-acetylneuraminic acid (NANA) [98]. A MIP shell was photopolymerized onto the QDs by using methacrylamide (MAM), ethylene glycol dimethacrylate (EGDMA), and eosin/TEA in the presence of target molecules (Figure 14a). Since the QDs possess tunable light emission and broad excitation features, they used the different light emissions for multiplexed cell imaging. Green (550 nm) and red (660 nm) light-emitting QDs were excited in the presence of a UV source. In vivo cell imaging studies showed that MIP-coated QDs provided better binding to human keratinocytes and can be used as a versatile multiplexed imaging tool (Figure 14b).

### 4.4. Bioseparation

Novel bioseparation techniques are in increasing demand, given the importance of identifying and efficiently removing proteins or biological compounds of interest from bodily fluids for disease diagnostics and treatment. Efficient separation of biomolecules, especially highly abundant proteins in proteomics, is required. Some inheriting gene mutations are responsible for producing abnormal types of proteins in the body. In this case, separating these highly abundant proteins from the potential biomarkers helps determine a disorder. MIP-based platforms are attractive materials for separation studies due to their easy fabrication, scale-up, and high stability [14,99,100].

**Figure 15:**
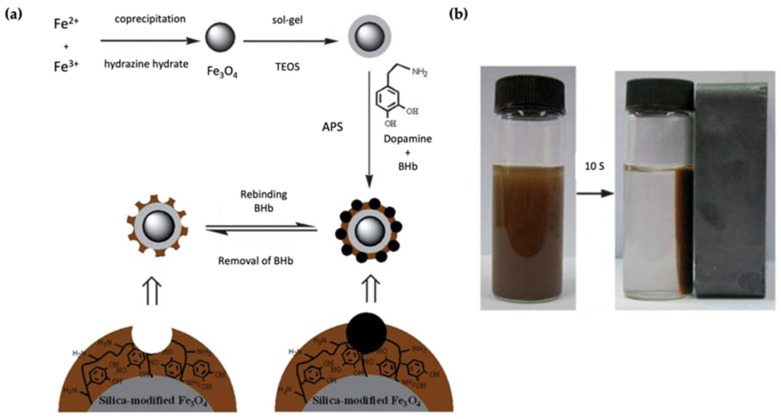
(**a**) General preparation of BHb imprinted SiO_2_@Fe_3_O_4_ nanoparticles, (**b**) magnetic response of the nanoparticles in the presence of an external magnetic field. Reproduce with permission from [102].

Polymer-coated magnetic nanoparticles are the perfect candidate for separation processes due to their facile separation by applying an external magnetic field [101]. Jia et al. demonstrated silica modified-Fe_3_O_4_ nanoparticles to separate bovine hemoglobin (BHb) [102]. Removal of the high abundance of BHb is essential to enrich the low abundance biomarkers and proteins. For this purpose, they coated polydopamine onto the SiO_2_@Fe_3_O_4,_ which avoided protein diffusion (Figure 15a). Surface imprinting was performed in the presence of the template molecule, dopamine, and ammonium persulfate (APS). Since the adsorption sites are close to the surface, the imprinted nanoparticles reached the adsorption equilibrium in a short time, and quick separation was achieved using a magnetic field (Figure 15b). BHb separation with polydopamine-coated inorganic nanoparticles was also demonstrated by Xia et al. [103]. They used SiO_2_ nanoparticles as the core material, which differed from the previously reported work. The protein recognition and separation profiles were determined by a single protein, BHb, or rebinding experiments in the presence of competitive compounds.

In another example, MIP core-shell superparamagnetic nanoparticles were prepared to separate cholesterol [104]. A high cholesterol level is associated with atherosclerosis, a chronic inflammatory disease within the artery wall [105]. The current treatments are either risky or relatively limited. Therefore, removing cholesterol using organic–inorganic hybrid systems is desirable as the reported design. A cholesterol imprinted core-shell nanoparticles were fabricated for this purpose. First, oleic acid-coated Fe_3_O_4_ nanoparticles were modified using a ligand exchange reaction with 3-(benzylsulfanylthiocarbonylsulfanyl) propionic acid (BSPA) to anchor BSPA onto the surface. Surface-mediated RAFT polymerization was employed for obtaining a cholesterol-imprinted polymer shell. The hybrid nanocomposites were obtained 20 nm in size with a 6 nm MIP shell. The final product was tested using human serum samples and successfully separated cholesterol with a recovery of 91.6%. The approach here can be modified to establish a separation system for any target steroid.

Recently, a multi-template imprinted polymer was combined with a hybrid nanocomposite system [106]. The selective separation and determination of nonsteroidal anti-inflammatory drugs, naproxen (NPX), methocarbamol (MTH), and omeprazole (OMZ) from biological and pharmaceutical samples was demonstrated. They utilized the composition of multiple nanomaterials to reinforce the final product’s performance. The imprinted polymer layer was constructed onto the Fe_3_O_4_/ZnO/CuO/MWCNT nanocomposite core. The presence of MWCNT provides high contact surface and electrical conductivity. Carbon paste electrodes were employed to obtain satisfactory analytical results for separating and detecting NPX, MTH, and OMZ.

## 5. Conclusions and Future Perspectives

In this review, we presented a general overview of the recent progress in the design and fabrication of MIP-coated inorganic nanoparticles and highlighted their applications in several areas of biomedical sciences. While in the past, MIPs were generally used in sensing applications because of their cheaper cost and higher stability as compared to their biological counterparts such as natural antibodies, advances in their synthesis and integration with functional nanomaterials have led to diverse applications.

Core-shell MIPs have displayed great potential in biomedical science due to their excellent characteristics, such as biocompatibility, stability, controlled size, and the efficient accessibility of the binding sites on the imprinted layer. Using inorganic nanomaterials such as Au, Ag, or Fe_3_O_4_ as a core material, helps the organic–inorganic system to have the capacity to respond to external physical stimuli. Therefore, Au, Ag, and Fe_3_O_4_ nanomaterials are perfect candidates for bioimaging, bio/chemosensors, and bioseparation applications. Examples illustrate that combining MIPs with QDs opens new doors in the bioimaging area. On the other hand, cytotoxicity is the main challenge for inorganic nanoparticles to be further used in in vivo studies. Even though many examples have been presented for in vitro and short-term in vivo studies, there is still a demand for more investigations to demonstrate lower toxicity in clinical uses. Since SiO_2_ nanoparticles indicate excellent biocompatibility, they can be employed for in vivo biological applications. SiO_2_-based coatings can be either directly imprinted or provide a layer to increase the biocompatibility of the inorganic core. Few investigations have been undertaken so far, suggesting that MIP-coated inorganic nanoparticles are suitable candidates for in vivo studies, thus indicating that they may be amenable for translation to clinical settings. The advantages and flexibility of the molecular imprinting technology, and rapid developments in the functionalization and modifications of core inorganic nanoparticles, convincingly suggest that MIP-coated inorganic nanoparticles will continue to play an increasingly important role in the fabrication of diagnostic tools and drug delivery devices.

## Figures and Tables

**Table 1:** Comparison of bulk MIPs and core-shell MIP nanoparticles.

Properties	Bulk MIPs	Core-Shell MIP NPs
Synthesis method	Easy	Complex
Morphology	Irregular	Uniform
Surface area	Smaller	Larger
Adsorption capacity	Lower	Higher
Binding sites distribution	Heterogeneous	Homogeneous
Template leakage	More	Less
Yield	Lower	Higher

**Table 2:** Summary of the presented works.

Target	Core Material	Linear Range	LOD	Ref.
Hemoglobin	Fe_3_O_4_@SiO_2_	0.005–0.1 mg/mL	0.0010 mg/mL	[76]
Pyocyanin	Fe_3_O_4_@SiO_2_	-	-	[77]
TNF-α	Fe_3_O_4_@SiO_2_	0.01 pM–100 nM	0.01 pM	[78]
Metronidazole	GQDs@SiO_2_	0.2–15 μM	0.15 μM	[82]
Dopamine	CdTe QDs@SiO_2_	0–1.2 × 10^−6^ M	(100–50) × 10^−9^ M	[83]
Dopamine	Au@SiO_2_	4 × 10^−8^–5 × 10^−5^ M	2 × 10^−8^ M	[85]
Carcinoembryonic Antigen	Au	-	0.1 ng/mL	[86]

## Data Availability

Not applicable.
